# Upfront molecular testing in patients with advanced gastro-esophageal cancer: Is it time yet?

**DOI:** 10.18632/oncotarget.4247

**Published:** 2015-06-10

**Authors:** Sameh Mikhail, Kristen Ciombor, Anne Noonan, Christina Wu, Richard Goldberg, Weiqiang Zhao, Lai Wei, Kristina Mathey, Melissa Yereb, Cynthia Timmers, Tanios Bekaii-Saab

**Affiliations:** ^1^ Department of Medicine, The Ohio State University Comprehensive Cancer Center-James Cancer Hospital and Solove Research Institute, Columbus, Ohio 43221, USA; ^2^ Department of Pathology, The Ohio State University Comprehensive Cancer Center-James Cancer Hospital and Solove Research Institute, Columbus, Ohio 43221, USA; ^3^ Department of Biostatistics, The Ohio State University Comprehensive Cancer Center-James Cancer Hospital and Solove Research Institute, Columbus, Ohio 43221, USA

**Keywords:** next generation sequencing, gastro-esophageal cancer, molecular profiling, c-MET, targeted therapy

## Abstract

**Introduction:**

Targeting HER2 has improved outcomes in metastatic GE (mGE) cancer. In this study, we aim to explore the feasibility of molecular profiling in patients with refractory mGE cancer in routine clinical practice.

**Methods:**

Archival formalin-fixed, paraffin-embedded (FFPE) samples for patients with mGE were analyzed with commercially available targeted next generation sequencing (NGS) and/or FISH for *MET* amplification. We also reviewed the patients' medical records for concurrent HER 2 testing.

**Results:**

Tumor samples from 99 patients with mGE cancer were analyzed as follows: NGS (*N* = 56), FISH for *MET* amplification (*N* = 65), IHC and/or FISH for HER2 (*N* = 87). Of patients who underwent NGS, 50/56 (89%) had at least one actionable molecular alteration. The most notable actionable alterations included cell cycle abnormalities (58%), *HER2* amplification (30%), *PI3KCA* mutation (14%), *MCL1* amplification (11%), *PTEN* loss (9%), *CDH1* mutation (2%) and *MET* amplification (5%). Ninety-two percent (12/13) of patients with *HER2* amplification by NGS were positive for HER2 by IHC and/or FISH. In contrast, only 12/18 (66%) patients positive for HER2 by IHC and/or FISH demonstrated *HER2* amplification by NGS.

**Conclusion:**

Comprehensive molecular testing is feasible in clinical practice and provides a platform for screening patients for molecularly guided clinical trials and available targeted therapies.

## INTRODUCTION

Gastro-esophageal cancers (GE) represent a substantial health care challenge in many parts of the world. The incidence of esophageal adenocarcinoma (EC) increased markedly in the United States and Western countries in recent decades [1–3]. Furthermore, gastric cancer is the world's third leading cause of cancer-related mortality in 2012, accounting for greater than 700, 000 deaths [4]. The poor prognosis of metastatic GE (mGE) malignancies and increasing frequency of esophageal cancer warrants increased efforts to further understand their biology and improve the available treatment options. EC develops from intestinal metaplasia of the esophageal epithelium, a condition known as Barrett's esophagus. Barrett's esophagitis develops as a result of chronic gastro-esophageal reflux disease (GERD) [5]. It has been hypothesized that chronic inflammation results in accumulation of molecular alterations that are associated with carcinogenesis [6]. In order to better understand pathogenesis of GE cancers, a limited number of studies have described molecular alterations in specimens obtained from patients with esophageal and gastric cancer [6, 7]. The information provided was helpful in understanding the genomic landscape and the spectrum of molecular alterations in GE cancers, but the impact of this information on clinical practice remains unclear. It became obvious that it was essential to bring the science of molecular biology from the bench to the bedside, given its potential benefit in introducing novel treatment strategies to the management of patients with GE cancer. Immunohistochemistry (IHC) and fluorescent in-situ hybridization (FISH) have played an integral role in identifying HER2 positive patients. Recently, the introduction of next-generation sequencing (NGS) has facilitated the characterization of molecular alterations and allowed for incorporation of molecular testing into routine clinical practice. Additionally, the use of IHC and FISH has expanded to identify novel biomarkers such as *MET* amplification. Herein we present our experience with using NGS and other molecular testing techniques in patients with mGE cancers in clinical practice to determine its feasibility and define the spectrum of actionable mutations in our population. To our knowledge, this is the first published report describing the use of NGS and other molecular profiling techniques for the molecular characterization of mGE tumors in routine clinical practice.

## RESULTS

**Figure 1A:**
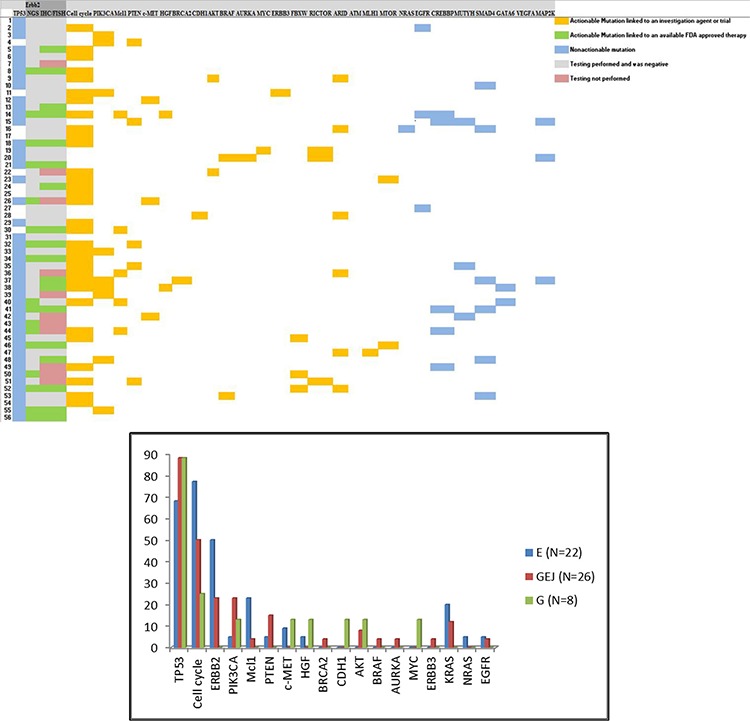
Distribution of genomic alterations in patients tested with next-generation sequencing **Blue:** Non-actionable mutations; **Orange:** Actionable mutations with available investigational agents; **Green:** Actionable mutation with available FDA approved therapy. **Figure 1B:** Distribution of molecular changes based on site of the tumor. **Vertical axis:** percentage of tumors that carry mutation of interest. **Horizontal axis:** Notable actionable and non-actionable mutations. **E:** Esophageal adenocarcinoma; **GEJ:** Gastroesophageal junction cancer; **G:** Gastric adenocarcinoma

Primary or metastatic tumor samples from 99 patients with metastatic GE adenocarcinoma were tested with NGS and/or FISH for *MET* amplification. The site of the primary tumor was as follows: 49 patients with distal esophageal, 34 with GE junction and 16 patients with gastric cancer. Fifty-six patients underwent NGS (Figure 1A) and 65 patients underwent FISH for *MET* amplification. Twenty-one patients had both tests performed. Testing for HER2 (IHC and/or FISH) was performed in 87/99 (88%) patients.

### Next generation sequencing

The 56 patients who underwent NGS included 22 patients with distal esophageal cancer (EC), 26 patients with cancer of the gastro-esohageal junction (GEJ) and 8 patients with gastric adenocarcinoma (G). Of those patients, 50 (89%) had at least one actionable molecular alternation. The median number of molecular alteration per patient was 4 (range 1–13). The median number of actionable alteration per patient was 1 (E = 1, GEJ = 2; G = 1; range 0–4). *TP53* was the most commonly observed mutation and was observed in 45/56 (80%) patients. The most common actionable alterations included (Figure 1B) cell cycle abnormalities (*CDKN2A* mutation, *CCNE* amplification, *CDK 4/6* amplification, and *CCND* amplification) in 32/56 (58%) patients, *HER2* amplification in 17/56 (30%), *PI3KCA* in 8/56 (14%), *MCL1* amplification 6/56 (11%), *PTEN* loss in 5/56 (9%), *AKT* amplification in 3/56 (5%), *c-MET* amplification in 3/56 (5%) and HGF amplification in 2/56 (4%) patients. Other notable mutations included *CDH1* and *BRCA2* mutations, each of which was found in one patient and through additional testing were found to be germline mutations.

### C-MET testing

In the whole cohort, 5/99 (5%) patients were positive for *MET* amplification with 3/56 (5%) cases detected by NGS and 3/65 (5%) cases detected by FISH. Twenty one patients had both NGS and FISH performed and the results showed 100% (21/21) concordance. One patient had *MET* amplification demonstrated by FISH and NGS, while 20 patients were *MET* non-amplified by both tests. Of 5 patients with *MET* amplification, four patients were screened for clinical trial participation but their condition declined rapidly after *MET* testing was ordered and were unable to receive any further therapy. One patient is currently being considered for c-MET directed therapy. Of note, NGS demonstrated concurrent *MET* and *HER2* amplification in one patient whose cancer progressed while receiving treatment on a clinical trial in the first line setting and did not receive MET or HER2 directed therapy.

### HER 2 testing

In the whole cohort, 35/99 (35%) patients were HER2 positive; 17/56 (30%) cases were detected by NGS and IHC and/or FISH were positive in 30/87 (34%) patients. Forty-five patients underwent NGS plus IHC and/or FISH for HER2 testing. Of those patients, 12/13 (92%) patients with *HER2* amplification by NGS were also positive for HER2 by IHC and/or FISH. In contrast, only 12/18 (66%) patients were positive for HER2 by IHC and/or FISH demonstrated *HER2* amplification by NGS. Twenty-six patients were negative for HER2 by both NGS and IHC/FISH. Overall concordance (Table 1) between NGS and IHC/FISH was 84% (38/45). It is worth noting that one of the patients had *HER2* amplification by NGS and was negative for *HER2* by FISH performed prior to the patient's referral to our institution. When FISH was repeated in our center, it was found to be positive and the patient was offered HER2-directed therapy.

**Table 1:** Concordance between next generation sequencing (NGS) and immunohistochemistry IHC and Fluorescent in situ hybridization for HER 2; *N* = 45; +ve: positive; −ve: negative

HER 2 testing	IHC/FISH +ve	IHC/FISH -ve
**NGS +ve**	12	1
**NGS -ve**	6	26

## DISCUSSION

In order to achieve the goal of personalized medicine in mGE cancers, it is important to understand the molecular landscape of GE tumors and to develop a testing platform that is feasible for use in routine clinical practice. Additionally, it is essential to continue to develop novel targeted agents that have favorable clinical activity and acceptable toxicity.

The TOGA study [8] has convincingly demonstrated that targeting HER2 in patients with gastric and GEJ cancers significantly improves survival with an acceptable side effect profile. Similarly, a recent report suggested that targeting *MET*-amplified refractory mGE cancer is associated with encouraging clinical activity. The objective response rate was 62%, which was very impressive for a pretreated population [Kwak E.; ASCO GI Cancer symposium 2015]. Additionally, several promising cell cycle inhibitors are currently under development. LEE011, a cyclin-dependent kinase (CDK) 4/6 inhibitor [Infante JS; ASCO Annual Meeting 2014] is under evaluation in a phase II study in patients with CDK 4/6 pathway activated tumors. Palbociclib, another CDK 4/6 inhibitor, was granted FDA approval in combination with letrozole with patients with estrogen receptor positive advanced breast cancer [9]. LY2835219 has also shown promising potential in early phase clinical trials [Patnaik AR; AACR Annual Meeting 2014; Wade Goldman JG; ASCO Annual Meeting 2014]. PI3KCA inhibitors are also showing encouraging results. Buparlisib (BKM120) was recently evaluated with paclitaxel in advanced breast cancer and demonstrated an acceptable safety profile and promising preliminary activity (Zambrano CS; ASCO Annual Meeting 2014]. It is clear now that the number of targeted therapies is increasing and that patients have more options for therapies that have promising activity against several molecular alterations if this testing is performed.

**Figure 2:**
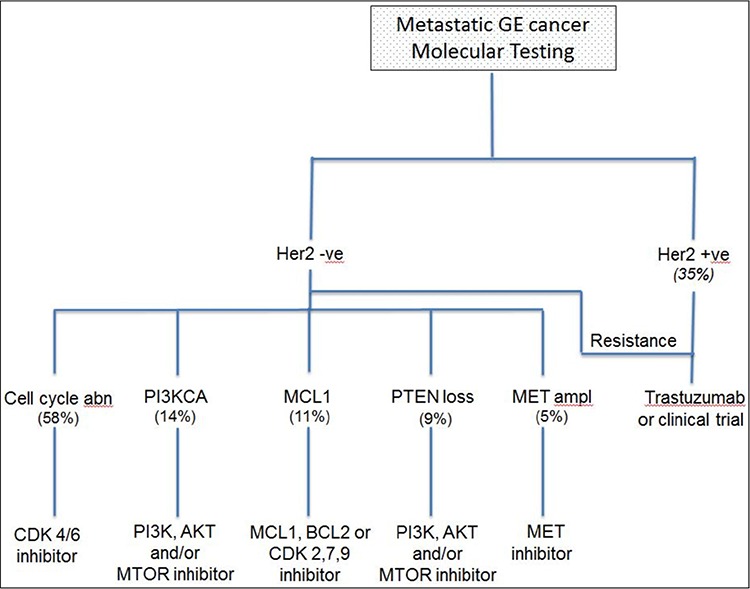
Proposed algorithm showing the use of molecular testing in clinical practice as a platform to direct patients towards relevant clinical trials Abn: Abnormalities (*CDKN2A* loss, *CCNE* amplification, *CDK 4/6* amplification and *CCND* amplification); amp: amplification. Italicized numbers repeat clinical trial identification numbers

A number of studies have described molecular alterations in gastric and esophageal tumors [6, 7]. The data was, however, generated in the research setting. While this information was very helpful in understanding the genomic landscape in GE cancer, its applicability to routine clinical practice was not addressed. In our report, we have used CLIA certified assays to better understand the frequency of actionable mutations in patients undergoing treatment for mGE cancers. Our results demonstrated that molecular testing is feasible in clinical practice and provide a platform for screening patients for molecularly targeted therapies. The most common molecular changes such as HER2, cell cycle protein dysregulation and PIK3CA can be targeted by several standard and promising investigational agents (Figure 2). Additionally, NGS can uncover familial mutations such as *CDH1* and *BRCA2* and allow providers to identify patients that need further testing for germline mutations. NGS, therefore, seems to offer a meaningful clinical benefit and is worth including in routine clinical practice.

The distribution of molecular alterations in our cohort was consistent with that observed by Dulak *et al* [6] in patients with esophageal cancer. The Cancer Genome Atlas Research Network [7] has proposed a new molecular classification in gastric cancer dividing it into four subtypes: 1) Tumors positive for Epstein-Barr virus (EBV) which display recurrent PIK3CA mutations among other mutations, 2) microsatellite unstable tumors which show elevated mutation rates including mutations of genes encoding targetable oncogenic signaling proteins, 3) genomically stable tumors which are enriched for diffuse histological variant of gastric cancer and 4) tumors with chromosomal instability which show marked aneuploidy and focal amplification of receptor tyrosine kinases. Our cohort validates the new molecular classification. A large proportion of patients (Figure 1) have chromosomal instability with frequent TP53 mutations. A smaller cohort of patients belongs to the EBV-positive group with recurrent PI3KCA mutations. A third small cohort demonstrated genomically stable tumors that carried a CDH1 mutation in one case. As our understanding of the genomic changes in mGE cancers increases, we hope that this classification will be translated into more specific clinical interventions.

The widespread use of molecular testing will also allow us to understand the effect of molecular alterations on the biology and behavior of GE tumors. As an example, in our cohort, patients with *MET* amplification progressed rapidly on chemotherapy and the condition of 4/5 patients deteriorated rapidly before they could receive MET directed therapy. This observation is consistent with a previous report that suggested that c-MET expression in gastric cancer was associated with more advance disease at presentation [10]. We, therefore, suggest testing for molecular targets early at presentation of the disease to better understand the disease biology and to allow for introduction of molecularly targeted therapy early in the course of treatment. This is particularly beneficial in cases where there is a limited window of opportunity for targeting molecular aberrations as is in the case of *MET* amplified mGE cancers.

Identifying molecular abnormalities offers the potential to direct patients towards treatment with novel agents that have activity against their molecular abnormalities. This was highlighted in a patient in our cohort with refractory esophageal cancer who progressed on 5-fluorouracil, platinum, irinotecan and taxane-based therapy. He was found to have a FBXW7 mutation and was started on everolimus which resulted in disease stabilization for > 6 months. Additionally, comprehensive molecular testing could identify mechanisms of resistance to targeted therapy. Our cohort included a patient with concurrent *MET* and *HER2* amplification as well as another patient with *HER2* amplification and PTEN loss. *MET* amplification [11] and PTEN loss [12] have both been described as potential mechanisms of resistance to HER2 targeted therapy and therefore knowledge of such molecular changes may prove useful in personalization of treatment and overcoming such mechanisms of resistance.

A few challenges will need to be addressed as molecular testing strategies become more integrated into the clinic. Cost effectiveness studies are needed to study the economic impact and feasibility of more widespread use of molecular testing and targeted therapy in the treatment of patients with mGE cancer. Commercial NGS testing is costly and occasionally not covered by insurance carriers, but we suspect that the cost of testing and targeted therapy will decrease with increase competition and decrease reagent cost to conduct the assays as they become more incorporated in routine clinical practice. Similarly, improving the clinical utility of NGS will require further research to identify the clinical and biological relevance of the mutations detected and to determine their predictive value when novel targeted agents are used. Studies are therefore ongoing to pinpoint *driver* mutations in GE cancer that can be effectively targeted with novel agents [6]. The feasibility of this concept was recently demonstrated in a report showing encouraging preliminary clinical activity of AMG 337 in patients with *MET* amplified advanced GE cancers [Kwak E.; ASCO GI Cancer symposium 2015]. Additionally, the turnaround time of NGS tests remains a challenge [13]. In our cohort, results were available approximately three to four weeks after ordering NGS. This timeline is less than ideal if treatment decisions need to be made promptly. In order to improve the utility of NGS and other molecular technologies, it will be essential to improve the turnaround time to less than two weeks. In our practice, we order NGS as part of the initial evaluation of patients with GE cancer. This allows for early identification of genomic alterations but given the suboptimal turnaround time this information often is not used in making first line treatment decisions. Furthermore, as chemotherapy can induce additional genomic alterations in tumors [14, 15], ideally NGS should be tested on recently obtained biopsy specimens. We believe again that use of this data will be only feasible if the turnaround time of NGS significantly improves. The concordance between NGS and IHC/FISH testing for HER 2 was less than optimal in our cohort (84%) although it is important to note that IHC/FISH and NGS were not systematically conducted on the same specimen. Previous studies have demonstrated high concordance (> 95%) between NGS and IHC/FISH testing for HER2 in breast cancer samples [16]. To our knowledge, concordance between NGS and IHC/FISH has never been evaluated in GE cancers however, a previous study suggested that IHC/FISH and NGS exhibited poor concordance for c-MET testing in a large cohort of patients with various tumors including GE cancers [17]. The suboptimal concordance observed in our cohort may be related to tumor heterogeneity or sampling errors, both of which are commonly encountered challenges in HER2 testing in patients with GE cancers [18, 19]. Nevertheless, it will be important to further evaluate the concordance between NGS andother molecular testing platforms in the clinical setting to determine if FISH and IHC testing are still needed in patients who undergo NGS. Until the concordance of these assays is further clarified, it is reasonable to use multiple technologies to evaluate HER2 and c-MET status in patients with mGE cancer. Finally, it is important to also note that alterations such a BRCA or CDH1 detected by NGS may represent somatic rather than germline mutations. Patients with such mutations detected by NGS will require further evaluation to define their clinical relevance. Nevertheless, NGS offers the potential for using it as a diagnostic tool for analysis of genetic mutations such as BRCA [20].

Our knowledge and understanding of the relevance of genomic alterations is still in its infancy. As more mutations are being identified and novel targeted agents are introduced the “actionability” of mutations may increase over time. In our report, we categorized mutations as actionable if they were linked to a standard therapy as in the case of HER 2 mutations or render patients eligible for a molecularly targeted clinical trial. As an example, PI3KCA mutations and PTEN loss were included as actionable alterations given the availability of a trial (NCT 01430572) of pozapanib and everolimus in PI3KCA mutation positive/PTEN loss patients as well as trials evaluating the role of the PI3KCA inhibitor, BKM120 (NCT01297452). MCL1 was also considered an actionable mutation since patients were referred to a clinical trial (NCT01303341) of riluzole and sorafenib in patients with advanced solid tumors and melanoma [21]. Similarly, in our institution, patients with alterations in CDKN2A, CDK 4/6 or CCND1–3 amplification were offered participation in a clinical trial (NCT01037790) investigating the benefit of the CDK 4/6 inhibitor LEE011 in patients with these alterations. It is worth noting that the value of these mutations as predictive markers may be redefined with further research and clinical trials. We believe, however, that NGS will continue to be an important tool in analyzing molecular alterations and identifying their clinical relevance.

In summary, our report demonstrated that comprehensive molecular testing with available NGS technologies is feasible and possibly beneficial in clinical practice. It provides a platform to direct patients towards novel molecularly targeted therapies that have very promising potential and to identify potential mechanisms of resistance to such therapies.

## PATIENTS AND METHODS

We retrospectively reviewed records of 99 patients with metastatic mGE adenocarcinoma who underwent molecular testing between January 2012 and February 2015 at the Ohio State University-James Cancer Hospital (OSU). All molecular testing was performed in Clinical Laboratory Improvement Amendments (CLIA)-certified laboratories and included a targeted NGS panel and FISH for *MET* amplification. We also reviewed the patients' medical records for concurrent testing for *HER2* amplification by FISH or HER2 overexpression by IHC. The conduct of this study was approved by the Ohio State University Medical Center Institutional Review Board.

### Next generation sequencing

Available formalin-fixed paraffin-embedded tissue (FFPE) from biopsies of primary or metastatic tumor sites was sent for targeted NGS at Foundation Medicine (Cambridge, MA). The test simultaneously sequences the entire coding sequences of 236 cancer-related genes, 7 introns from 19 genes often rearranged or altered in cancer to an average depth of coverage of greater than 250 times. The methodology of Foundation One NGS has been previously described in detail [22–25]. Genomic alterations were categorized as “actionable” if they were linked to an approved, standardly available, investigational therapy or would result in a change in the management of the patient.

### Fluorescent in-situ hybridization (FISH) for *MET* and *HER2* amplification

Amplification of the *MET and/or HER2* gene was evaluated with FISH on tumor containing 4 micron FFPE sections using a combined chromosome 7 centromeric probe (CEP7, Abbott Labs) and synthesized *MET* DNA Probe from BAC clone (Roswell Park Cancer Institute) that spans the entire *MET* gene on chromosome 7 or PathVysion HER2 DNA Probe Kit consisting of a chromosome 17 centromeric probe (CEP17) and a separate HER2 probe that spans the entire gene. Slides/sections were scanned and analyzed using a validated semi-automated scanning imaging workstation and accompanying imaging analysis software (Bioview). Slides/sections were then reviewed manually by a pathologist. The classification of tumor to a *MET or HER2* positive [*MET/HER2* (+)] or *MET/HER2* negative [*MET/HER2* (−)] class is based on the determination of the copy numbers of *MET/HER2* gene versus CEP7. In a nucleus with a normal copy number of the *MET* gene, two red signals will be observed. Abnormal copy number of the *MET* gene is indicated by more than two copies of the red probe signal with a ratio of *M*ET/CEP7 > 2.3. An equivocal result will have a ratio 1.8–2.2. In contrast, a ratio < 1.8 will be considered as negative for *MET* amplification. Similarly, a positive result for HER2 gene amplification is rendered when the majority of invasive tumor cells meet any of the following criteria: 1) HER2/CEP17 ratio > = 2.0 with an average HER2 copy number > = 4.0 signals per cell, 2) HER2/CEP17 ratio > = 2.0 with an average HER2 copy number < 4.0 signals per cell, or 3) HER2/CEP17 ratio < 2.0 with an average HER 2 copy number ≥ 6.0 signals per cell.

### HER2 overexpression

HER2 protein expression in gastric and GEJ adenocarcinomas was evaluated by IHC on FFPE tissues, using clone 4B5 (rabbit monoclonal, Ventana) on a Ventana auto-stainer. Membrane staining of tumor cells is evaluated and graded as follows: 0 (negative), no immunoreactivity (biopsy) or membranous immunoreactivity in < 10% of tumor cells (resection); 1+ (negative) tumor cell cluster (5 cells) with faint immunoreactivity regardless of percentage of cells stained (biopsy) or faint staining in > 10% of tumor cells but only a portion of the membrane is positive (resection); 2+ (equivocal), tumor cell cluster with weak to moderate complete, basolateral or lateral membrane immunoreactivity regardless of percentage of cells (biopsy) or complete, basolateral or lateral membrane staining in > 10% of tumor cells (resection); 3+ (positive), tumor cell cluster with strong complete, basolateral or lateral membrane staining regardless of percentage of cells stained (biopsy) or strong complete, basolateral, or lateral membrane staining in > 10% of cells (resection). Equivocal (2+) staining triggers reflexive FISH testing.
